# First Appearance

**Published:** 1883-07

**Authors:** 


### FIRST APPEARANCE.

First almanac printed in 1460.

Envelopes were first used in 1839.

The first steel pen was made in 1830.

The first air pump was made in 1654.

The first lucifer match was made in 1798.

Mohammed was born at Mecca about 570.

The first iron steamship was built in 1830.

The first balloon ascent was made in 1798.

Coaches were first used in England in 1569.

The first horse railroad was built in 1826.

Gold was discovered in California in 1848.

The first steamboat plied at Hudson in 1807.

The entire Hebrew Bible was printed in 1488.

The first telescope was used in England in 1608.

Christianity was introduced into Japan in 1549.

The first watches were made at Nurenburg in 1477.

The first newspaper advertisement appeared in 1652.

Percussion arms were used in the U. S. army in 1830.

The first use of a locomotive in this country was in 1829.

Kerosene was first used for lighting purposes in 1826.

The first copper cent was coined in New Haven in 1687

The first glass factory in the United States was built in 1780.

The first printing press in the United States was worked in 1620.

Glass windows were first introduced into England in the eighth century.

The first steam engine on the continent was brought from England in 1753.

The first complete sewing machine was patented by Elias Howe, Jr., in 1846.

The first daily newspaper appeared in 1702. The first newspaper printed in the United States was published in Boston on Sept. 25, 1790.

The manufacture of porcelain was introduced into Hezin, Japan, from China in 1513. And Hezin ware still bears Chinese marks.

The first telegraphic instrument was successfully operated by S. F. B. Morse, the inventor, in 1835, though its utility was not demonstrated to the world until 1842.

